# Thermal and Mechanical Properties of Biocomposites Based on Polylactide and Tall Wheatgrass

**DOI:** 10.3390/ma16216923

**Published:** 2023-10-28

**Authors:** Cezary Gozdecki, Krzysztof Moraczewski, Marek Kociszewski

**Affiliations:** Faculty of Materials Engineering, Kazimierz Wielki University in Bydgoszcz, ul. Chodkiewicza 30, 85-064 Bydgoszcz, Poland; gozdecki@ukw.edu.pl (C.G.); kocisz@ukw.edu.pl (M.K.)

**Keywords:** biocomposite, PLA, hemp, tall wheatgrass, mechanical properties, DSC, DMA, TG

## Abstract

Biocomposites based on polylactic acid (PLA), tall wheatgrass (TWG), and hemp (H) were made by injection molding. The article discusses the impact of the agrofiller content on the composite properties, including thermal (DSC, DMA, and TG) and mechanical characteristics (tensile modulus, tensile strength, and impact strength). Generally, the introduction of a plant filler into the polylactide matrix reduced the thermal resistance of the resulting composites. Plant fillers influenced primarily the cold crystallization process, probably due to their nucleating properties. The addition of fillers to the PLA matrix resulted in an increased storage modulus across all tested temperatures compared to pure PLA. In the case of a composite with 50% of plant fillers, it was almost 118%. The mechanical properties of the tested composites depended significantly on the amount of plant filler used. It was observed that adding 50% of plant filler to PLA led to a twofold increase in tensile modulus and a decrease in tensile strength and impact strength by an average of 23 and 70%, respectively. It was determined that composites incorporating tall wheatgrass (TWG) particles exhibited a slightly elevated tensile modulus while showcasing a marginally reduced strength and impact resistance in comparison to composites containing hemp (H) components.

## 1. Introduction

Despite society’s growing awareness of the detrimental environmental effects associated with traditional plastics production, more than 98% of all plastics continue to be derived from fossil fuels, predominantly oil and gas. Recent data reaffirm that global plastics production in 2021 saw a notable rise compared to previous years, surpassing 390 million tons. This underscores the substantial and ongoing demand for plastics [1,2]. Hence, there is an urgent requirement for concerted efforts to decrease the manufacturing of polymers and composites originating from fossil resources. Therefore, one of the most important directions in environmental protection is replacing traditional petroleum-based plastics with new, biodegradable materials [3,4,5,6,7,8,9,10]. One of the most famous types of biodegradable composites are composites containing wood raw material—wood–plastic composite (WPC). The WPC is usually produced based on the basic thermoplastic polymers, e.g., polypropylene, polyethylene, polyvinyl chloride, etc., and wood particles [11,12,13,14,15,16,17,18,19,20,21]. Biodegradable and natural polymers are gaining significant interest in scientific circles and an increasing one in the industrial sector due to their environmentally friendly composting properties. To support a sustainable market economy and address current environmental challenges, biodegradable materials should play an increasingly significant role [22,23]. One of the basic and most frequently used polymers of natural origin and completely biodegradable is polylactic acid (PLA). It can be used without filling or be filled with raw materials, e.g., plant fibers. In this case, a completely biodegradable composite called a biocomposite is obtained. The possibilities of using PLA for the production of biocomposites have been studied many times [24,25,26,27,28,29,30]. Traditional WPC is manufactured using lignocellulosic material in the form of shredded wood. Apart from the basic form of wood raw material, which is wood flour [31,32,33], larger wood chips have also been used as a filler. Additionally, it has been shown that raw material from the wood industry is useful for the production of WPC [34,35,36]. One of the clear trends currently occurring in the world of composites is the replacement of reinforcing synthetic fibers by natural fibers. This is, among other things, due to the good availability, high specific strength, and low cost of natural fibers [6,37,38,39,40,41]. Although, traditional plant fibers have found wide application in the textile, paper production, and packaging industries, their unique properties are increasing their popularity in the production of biodegradable composite materials [10]. Extensive research has been conducted to explore the potential of various natural fibers for reinforcing composites. In numerous instances, test results have revealed that composites incorporating natural fibers offer numerous advantages compared to those using synthetic fibers. These benefits encompass an abundant availability, a renewable source of raw materials, a full biodegradability, and a high strength-to-weight ratio [9,42,43,44,45,46,47,48,49,50,51]. However, it should be remembered that the properties of composites containing natural fibers depend on many factors. One of the basic factors influencing the properties of a biocomposite is the content of the natural filler [52,53]. However, for various reasons, including processing problems [7], low filling levels are usually used. In the case of biocomposites filled with plant fibers such as hemp, linen, or jute, the amount of filler usually does not exceed 30% [54,55]. Nonetheless, it has been shown that it is possible to produce composites based on PLA and, for example, hemp, in which the content of plant raw material is 40% [50,56,57,58]. However, few studies indicate the efficient filling of PLA with hemp fibers at a level of 50% or higher [59]. But there are also known studies in which the content of natural fibers is below 10%. This is often due to inappropriate analyses being carried out [60]. In general, the connection between the reinforcing fiber and the matrix plays a crucial role in the mechanical properties of composites. Weak bonding between these two phases results in low mechanical and physical properties of the composite [61]. Many chemicals have been tested in laboratory experiments for their ability to improve the fiber/matrix interface of polymer composites, such as hydroxide, sodium peroxide, organic and inorganic acids, silane, acrylic anhydrides and monomers, and others. A beneficial effect of this modification was demonstrated, especially on the mechanical properties of composites with modified fiber [60,61,62,63]. It can be noted, however, that a commonly used method of preparing plant fibers is the modification of this raw material with NaOH. The topic of using various plants in the production of biocomposites is generally well documented. Often, the choice of a particular raw material is influenced by geographical factors or price. One promising approach to creating cost-effective biocomposites is using fast-growing raw materials. However, there is a lack of reports on the potential use of tall wheatgrass as a PLA filler for the production of biocomposites. This material or its variants have shown promise in the production of wood-based panels [64,65,66]. Although it is also mentioned in the context of other applications [8], it has not been sufficiently demonstrated whether tall wheatgrass or a high content of this filler can be used as a PLA filler. This type of biocomposite has the potential for a broad spectrum of uses. They can undoubtedly be used in many sectors, e.g., the packaging industry, for disposable products and housings of electronic products, in the furniture industry, and many others. Hence, the primary objective of this study was to explore the feasibility of incorporating tall wheatgrass as a reinforcing agent in PLA and to assess the characteristics of the resulting biocomposite composed of these two components. For comparisons, we also fabricated and evaluated biocomposites consisting of PLA and hemp fibers.

## 2. Materials and Methods

### 2.1. Materials

In the studies, two types of lignocellulosic plant material, tall wheatgrass (*Agropyron elongatum*) (TWG) and hemp (*Cannabis sativa* L.) (H) particles were used. Plant particles were produced by grinding whole stems, without separating the fiber fraction. Technical raw plant fibers (length 5 mm, dimension 0.25 mm) were obtained from Research & Development Centre for Wood-Based Panels Sp. z o. o. in Czarna Woda. The fibrous components were additionally screened (through an analytical LAB-11-200/UP sieve shaker using 35 mesh sieves) to obtain a repeatable material. As a matrix, the PLA 2003D was used in a granule form obtained from NatureWorks LLC. Minnetonka, MN, USA with a density of 1240 kg/m^3^ and melt flow index of 5.92 g/10 min (190 °C/2.16 kg). The TWG and H were treated with 5% NaOH to remove surface impurities, then washed using distilled water and dried to a moisture of 6%. The raw materials were mixed in proper proportions as shown in Table 1.

### 2.2. Samples Preparation

Test specimens were made by injection molding using a screw injection molding machine Wh-80 Ap. The temperature profile was 160, 170, and 180 °C. The injection pressure time, hold pressure time, and cooling time were 3, 6, and 40 s, respectively. The specimens were made according to EN ISO 527-2 [67]. After processing, specimens were stored under controlled conditions (50% relative humidity and 23 °C) for 2 weeks before testing.

### 2.3. Methods

#### 2.3.1. Thermogravimetric Analysis

Thermogravimetric analyses were performed in a nitrogen atmosphere using a Q500 thermobalance (TA Instruments, New Castle, DE, USA). Approx. 33 mg (composites) or 11.5 mg (hemp and tall wheatgrass) of samples was tested in the temperature range from 25 to 700 °C with a temperature change rate of 10 °C/min. The T_5%_ values corresponding to the temperature of the loss of 5% of the initial sample mass, humidity (M), and residue (R) were determined from the thermogravimetric curves. The value of T_5%_ was taken as a parameter determining the thermal resistance of the material.

#### 2.3.2. Differential Scanning Calorimetry (DSC)

Differential scanning calorimetry studies were performed in a nitrogen atmosphere using a Q200 scanning calorimeter (TA Instruments, New Castle, DE, USA). Approx. 12.5 mg of samples was tested in the temperature range from 0 to 200 °C. The temperature change rate was 10 °C/min. The glass transition temperature (*T_g_*), cold crystallization temperature (*T_cc_*), enthalpy change in the cold crystallization process (Δ*H_cc_*), melting point (*T_m_*), and enthalpy change in the melting process (Δ*H_m_*) of individual samples were determined based on the 2nd heating curve. The degree of crystallinity (X_c_) was calculated based on Formula 1, assuming that the enthalpy change value of 100% crystalline PLA (Δ*H_m_*_100%_) was 93 J/g [68].

(1) $$X_{c}=\left(\frac{H_{m}-H_{cc}}{H_{m100\%}\cdot \left(1-x\right)}\right)\cdot 100\%$$

where:

Δ*H_m_*—enthalpy change in the melting process;
Δ*H_cc_*—enthalpy change in the cold crystallization process;
Δ*H_m_*_100%_—enthalpy change value of 100% crystalline PLA;
*x*—filler content.

Thermomechanical tests (DMA) were performed using a Q800 dynamic mechanical analyzer (TA Instruments, New Castle, DE, USA). The tests were carried out in the temperature range from 30 to 150 °C with a heating rate of 3 °C/min. The samples had the shape of a cuboid with dimensions of 80 mm × 10 mm × 4 mm. The strain was 0.01% and the strain frequency was 1 Hz.

#### 2.3.3. Mechanical Testing

Mechanical properties of tested composites were evaluated in relation to tensile and impact properties. Tensile tests were performed according to EN ISO 527-2 using an Instron 3367 machine. The speed of the crosshead was 2 mm/min. Unnotched Charpy impact strength tests were conducted according to EN ISO 179-1 [69]. All tests were performed at room temperature (23 °C) and constant relative humidity (50%).

### 2.4. Statistical Analysis

The obtained data were statistically analyzed using Statistica version 13. A one-way analysis of variance (ANOVA) was conducted to determine the significance of the effect of the kind and natural filler content on the WPC mechanical properties. Tukey’s test was applied to evaluate the statistical significance between the mean values of the properties of composites with different fillers, respectively. The same letters indicate that there is no significant difference (at α = 0.05) for a given property between compared composites with different kinds of composites.

## 3. Results

Table 2 shows the TG results of the tested materials. The temperature value of the loss of 5% of the initial mass (T_5%_) was assumed as the thermal resistance of the materials.

The T_5%_ value of pure PLA was 310.2 °C. The introduction of the plant filler into the matrix resulted in a decrease in the thermal resistance of the obtained composites. The T_5%_ of the composite containing 10 wt.% of hemp dropped to 299.5 °C. The use of tall wheatgrass resulted in an even greater reduction in the thermal resistance of the produced composites. The obtained T_5%_ value of the TWG_90_10 sample dropped to 289.6 °C, so it was 10 °C lower than that of the H_90_10 sample.

**Figure 1:**
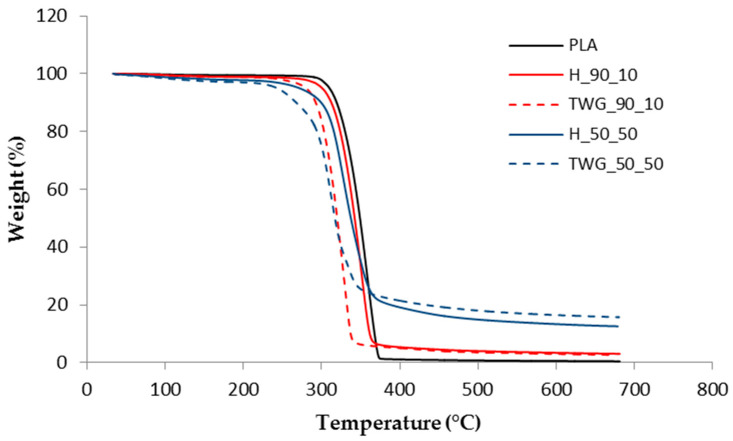
TG curves of selected samples.

Increasing the content of plant fillers resulted in an even greater decrease in the thermal resistance of composites (Figure 1).

With a maximum filler content of 50 wt.%, the T_5%_ value of the H_50_50 sample was 274.1 °C, and therefore, it was 36 °C lower than the value obtained for pure PLA. As the fiber content increased, the differences in the thermal resistance of composites with different fillers increased even further. Composites containing tall wheatgrass had a significantly lower thermal resistance compared to composites containing hemp. The T_5%_ value of the TWG_50_50 sample was only 243.9 °C, so it was 30.2 °C lower than the sample containing the same amount of hemp and as much as 66.3 °C lower than pure PLA.

The introduction of plant fillers in the composites caused changes in thermal resistance and differences in TG curves compared to PLA. An additional weight loss was observed at temperatures up to 100 °C, which was caused by moisture in the fillers. The weight loss due to moisture evaporation increased with an increasing filler content from 0.5% to 2%. This is a typical relationship: the more filler, the more water, and the greater the weight loss. The occurrence of a small amount of moisture in materials containing plant fillers is typical for this type of material due to the hydrophilic nature of the fillers used. Additionally, there were no significant differences between hemp and tall wheatgrass, which shows that the moisture absorption of these materials is at a similar level.

The introduction of fillers also resulted in the appearance of residues (R), i.e., ash resulting from the degradation process. As expected, the more filler was in the composite, the higher the R value. With filler content up to 30 wt.%, there were no significant differences between hemp and tall wheatgrass. The recorded amounts of R ranged from approx. 3% for materials containing 10 wt.% of filler up to approx. 8% for materials containing 30 wt.% of filler. Only at the highest filler content was the difference in residue noticeably greater for the composite containing tall wheatgrass (15.7% vs. 12.6%).

The reduction in the thermal resistance of composites compared to pure PLA is the result of the introduction of plant fillers into the polymer matrix. Fillers have significantly lower degradation temperatures compared to the PLA polymer (Table 3). Studies have shown that hemp begins to degrade at 285.9 °C and tall wheatgrass at 240.5 °C, which are much lower values than in the case of PLA (310.2 °C).

**Figure 2:**
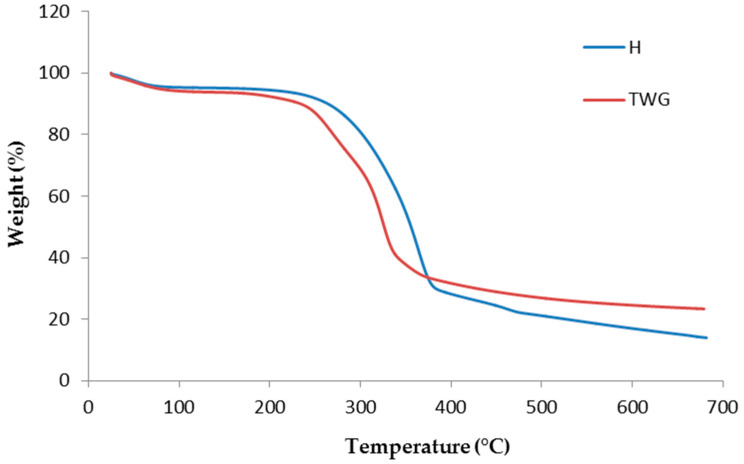
TG curves of H and TWG.

The degradation of composites showed differences due to the distinct degradation curves of hemp and tall wheatgrass (Figure 2). Initially, the course of mass change (up to 100 °C) was similar, which confirmed the previous statement about a similar moisture absorption of these two materials. Above this temperature, differences began to occur. The different nature of the degradation curves of hemp and tall wheatgrass at higher temperatures explains the differences observed in the degradation of the composites (Figure 2). The lower T_5%_ values of TWG samples resulted from the much lower thermal resistance of tall wheatgrass than hemp. There were more residues of the degradation process in tall wheatgrass, which was reflected in the R value results. This was especially noticeable with the highest filler content.

Table 4 shows the DSC results of the tested materials. The analysis was carried out based on the second heating curves. The first heating scans were aimed at removing the thermal history of the materials resulting from the processing process.

It can be concluded that the changes observed in the DSC curves were primarily influenced by the content of plant fillers and not their type. Therefore, it could not be indicated which of the tested fillers had a more significant impact on the thermal characteristics of the tested composites. Therefore, since there were no differences between materials containing hemp or tall wheatgrass, a further analysis is appropriate for both types of composites.

The glass transition temperature (*T_g_*) of the tested materials was practically the same regardless of the amount of plant filler in the PLA matrix. Although a slight decrease in the Tg values could be observed depending on the filler content, the observed changes were very small and did not affect the functional properties of the obtained composites. The observed slight decreases in *T_g_* may be the result of a faster heat transfer into the polymer phase of the composite associated with a smaller amount of the polymer phase. Reducing the polymer content resulted in a lower thermal lag and a lower recorded glass transition temperature during the thermal analysis of polymers.

**Figure 3:**
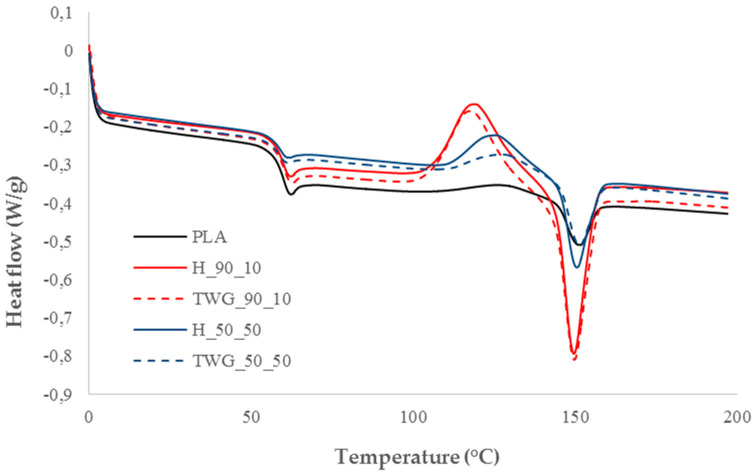
DSC curves of selected samples.

Plant fillers significantly impacted the cold crystallization and melting processes (Figure 3).

The introduction of the smallest quantity of fillers into the matrix greatly intensified the cold crystallization process, while simultaneously shifting the process towards lower temperatures. The enthalpy change in the cold crystallization process (Δ*H_cc_*) increased significantly from 4.7 J/g for pure PLA to approx. 21 J/g for composites containing 10 wt.% of fillers. At the same time, the cold crystallization temperature (*T_cc_*) decreased from 127.5 °C to approximately 119 °C. The consequence of increasing the intensity of the cold crystallization, and therefore increasing the amount of the crystalline phase formed, was an increase in the intensity of the melting process in which the crystalline phase melted. The enthalpy change in the melting process (Δ*H_m_*) increased from 5.1 J/g for pure PLA to approximately 22 J/g for composites containing 10 wt.% of fillers. However, the melting temperature (*T_m_*) changed slightly, decreasing from 151.1 °C to 149 °C. The structure of the crystalline phase of these materials was therefore practically the same, and since the changes in *T_m_* were similar even in the case of larger amounts of fillers, this also applied to the remaining composites.

Increasing the filler content led to a gradual decrease in the intensity of the cold crystallization and melting processes. With the increase in the quantity of fillers, the Δ*H_cc_* values decreased to approximately 15 J/g for composites containing 30 wt.% of fillers and 7–10 J/g for composites containing 50 wt.% of fillers. *T_cc_* values also increased to approximately 122 and 127 °C, respectively. As expected, the Δ*H_m_* values also decreased to approximately 15 J/g for a content of 30 wt.% of fillers and 7–11 J/g for 50 wt.% of fillers.

The observed large increase in the intensity of the cold crystallization and decrease in its temperature at a low filler content is probably caused by the nucleating properties of the fillers. Small filler particles serve as nucleating centers, increasing the growth of the crystalline phase. The decrease in intensity with higher filler contents may result from two phenomena. In the first case, an increase in the amount of filler may limit the growth of the crystalline phase due to the lack of space for the growth of crystallites, which are blocked by the filler particles. In the second case, the decrease in the intensity of cold crystallization may result simply from the lower content of the polymer phase in the tested composite, which translates into lower energy effects of the cold crystallization and melting processes.

Despite the very large impact of the fillers used on the cold crystallization and melting processes, DSC tests showed that the initial degree of crystallinity of all composites was lower than 2%. This is because the entire crystalline phase melting during the melting process was formed only in the cold crystallization process and was not present in the material before the test, which was confirmed by the similar values of Δ*H_cc_* and Δ*H_m_*.

Plant fillers also changed the thermomechanical characteristics of the composites. The introduction of fillers into the matrix caused an increase in the storage modulus (*E*’) in the entire tested temperature range compared to pure PLA (Table 5).

The E’30 value of pure PLA was 2625 MPa and increased significantly after adding plant fillers. The observed stiffening effect is typical for this type of composites, due to the much higher storage modulus of plant fillers compared to the storage modulus of polymers [2,3]. Adding 10 wt.% of fillers resulted in an increase in E’30 to 3149 for hemp and 3047 MPa for tall wheatgrass. After adding 50 wt.% of fillers, the E’30 values increased to 5427 MPa and 5716 MPa, respectively. This dependency was valid also for modules determined at higher temperatures.

Comparing hemp and tall wheatgrass, it can be concluded that up to 30 wt.% of filler, composites containing hemp were characterized by slightly better thermomechanical properties. At higher filler contents, especially at lower temperatures, composites with tall wheatgrass had an advantage.

**Figure 4:**
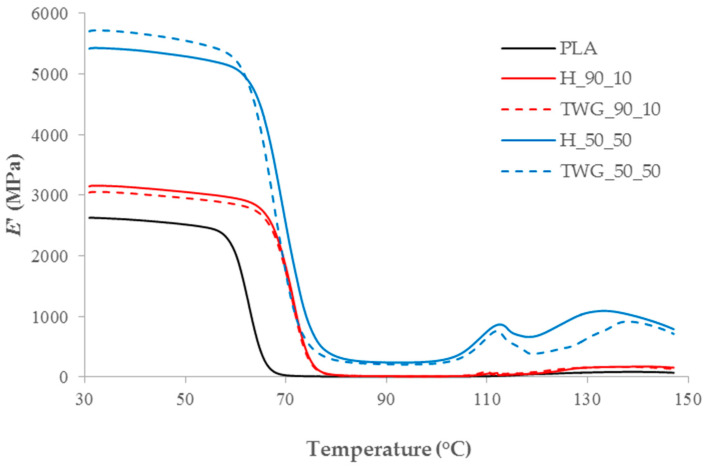
DMA curves of selected samples.

Noteworthy are the large differences in the E’130 value between individual composites (Figure 4).

The observed increase in E’130 was the result of the cold crystallization process. The crystallites formed in this process were characterized by much higher values of storage modulus, which translated into recorded changes. Interestingly, unlike DSC tests, samples containing 50 wt.% of fillers were characterized by a greater intensity of the cold crystallization process. This may therefore suggest that the decrease in the intensity of the cold crystallization observed in the DSC curves was mainly caused by a decrease in the share of the polymer phase in composites containing larger amounts of fillers.

### Mechanical Properties

**Figure 5:**
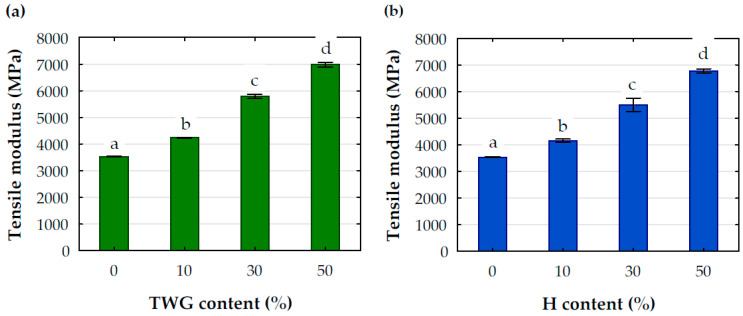
Tensile modulus of tested composites with (**a**) TWG and (**b**) H; a, b, c, d-statistical descriptors; values marked with the same letters are not statistically different (*p* > 0.05).

**Figure 6:**
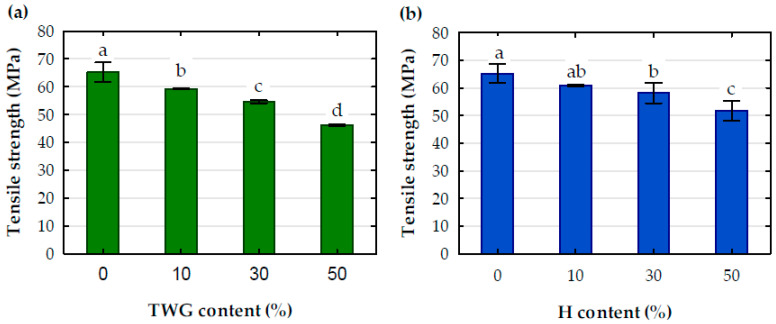
Tensile strength of tested composites with (**a**) TWG and (**b**) H; a, b, c, d-statistical descriptors; values marked with the same letters are not statistically different (*p* > 0.05).

**Figure 7:**
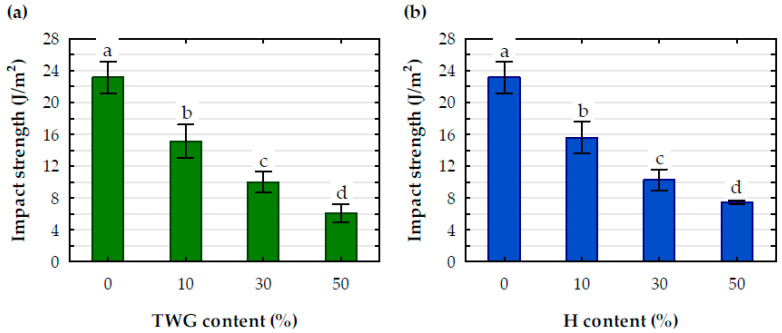
Impact strength of tested composites with (**a**) TWG and (**b**) H; a, b, c, d-statistical descriptors; values marked with the same letters are not statistically different (*p* > 0.05).

Figure 5, Figure 6 and Figure 7 present the results of testing the mechanical properties of the analyzed composites. The tensile modulus (Figure 5), regardless of the type of filler, significantly depended on the degree of filling of the composite. Adding 10 wt.% of filler to PLA caused an increase in the tensile modulus by an average of 19.7% and 17.5% when the filler was tall wheatgrass and hemp particles, respectively. Increasing the filling level from 10 to 30 wt.% caused an increase in the tensile modulus for both types of particles by an average of 34.5%, and after adding 50% of the filler, the increase in the modulus was even greater and amounted to an average of 64%. It is worth noting that PLA with 50 wt.% of hemp and tall wheatgrass particles resulted in an average twofold increase in the tensile modulus compared to the pure polymer.

The effect of the amount of filler on the tensile strength (Figure 6) was not as significant as on the tensile modulus. As expected, the tensile strength decreased as the degree of filling the polymer with plant particles increased, and the changes in strength values for the assumed filling levels, as well as for the tensile modulus, were generally statistically significant. This impact also depended on the type of filler and was much smaller for a composite containing hemp. The composite containing 10 wt.% of plant filler had an average tensile strength of 60.1 MPa, which was 7.8% lower than pure PLA. Increasing the amount of filler to 30 wt.% resulted in a decrease in the strength of the composite with tall wheatgrass and hemp particles by 7.9 and 4.6%, respectively. Changing the filler amount to 50 wt.% increased these decreases to 22.1 and 14.9%, respectively. In relation to the pure polymer, the composite filled with 50 wt.% tall wheatgrass and hemp particles were characterized by a reduced tensile strength by 29.1 and 20.6%, respectively, which confirmed that adding plant particles to the polymer had a much greater impact on increasing its stiffness than to the decrease in its tensile strength.

A significant effect of the filler was also noted for the impact strength (Figure 7), which decreased significantly with an increase in filler amount. The differences between the average impact strength values for individual levels of PLA filling with plant particles were statistically significant. Filling the polymer with 10 wt.% of plant particles reduced the impact strength compared to pure PLA by an average of 33.8%. Increasing the amount of filler to 30 wt.% reduced the impact strength by approximately 56%, and when it was 50 wt.%, this decrease increased to approximately 71%.

The reduction in tensile strength and impact strength can be attributed to the formation of numerous voids resulting from the addition of the plant filler. The number of voids increased with the rise in filler content. This was likely due to the inadequate dispersion and adhesion of the hydrophilic natural fiber within the hydrophobic polymer matrix. The recorded reduction in mechanical properties is a typical effect of the introduction of plant fillers, which should be taken into account when planning the use of WPC materials [70,71].

The type of plant particle added to the polymer generally has little effect on the mechanical properties. Usually, the differences between the values for the composite with tall wheatgrass and hemp particles do not exceed a few percent, but they are often statistically significant (Table 6).

The most significant variations were observed in composites that contained plant raw material at the highest analyzed percentage. When comparing TWG and H, it was found that the tensile strength and impact strength were 11% and 19% lower, respectively, in the composites that contained TWG. No significant differences were observed in the impact strength of 10% and 30% and tensile strength of 30% between composites containing TWG and H.

## 4. Conclusions

1. The introduction of a plant filler into the polylactide matrix reduced the thermal resistance of the resulting composites. The decrease in thermal resistance was greater in the case of the composite containing couch grass. Additionally, it was observed that increasing the content of plant fillers resulted in a further decrease in the degradation temperature of the composites.
2. Plant fillers influenced primarily the cold crystallization process, probably due to their nucleating properties. After the introduction of plant filler particles, a significant increase in the intensity of the cold crystallization process was observed, along with a simultaneous decrease in the temperature of this process.
3. Plant fillers change the thermomechanical characteristics of composites. The introduction of fillers into the matrix caused an increase in the storage modulus in the entire tested temperature range compared to pure PLA.
4. Filling PLA with plant particles allows one to obtain a composite with increased stiffness and reduced strength and impact resistance.
5. The mechanical properties of the tested composites depended significantly on the amount of plant filler used. Increasing the amount of filler resulted in an increase in the elastic modulus and a decrease in the elastic modulus as well as a decrease in tensile strength and impact strength.
6. The mechanical properties of composites depended only slightly on the type of plant particles used to produce them. Composites containing TWG particles had a slightly higher elastic modulus and slightly lower strength and impact strength than composites containing H.

## Figures and Tables

**Table 1:** Experimental design.

Composite Code	Filler Content (%)	Filler
PLA	0	-
H_90_10	10	H
TWG_90_10	10	TWG
H_70_30	30	H
TWG_70_30	30	TWG
H_50_50	50	H
TWG_50_50	50	TWG

**Table 2:** Results of TG tests of the tested composites.

Sample	T_5%_ (°C)	M (%)	R (%)
PLA	310.2	0.0	0.0
H_90_10	299.5	0.5	3.0
TWG_P_90_10	289.7	0.5	2.7
H_70_30	286.9	0.8	8.0
TWG_70_30	259.2	1.0	8.4
H_50_50	274.1	2.0	12.6
TWG_50_50	243.9	1.6	15.7

**Table 3:** TG results of H and TWG.

Sample	T_5%_ (°C)	M (%)	R (%)
H	285.9	4.5	14.0
TWG	240.5	6.1	23.3

**Table 4:** DSC test results.

Sample	T_g_ (°C)	T_cc_ (°C)	ΔH_cc_ (J/g)	T_m_ (°C)	ΔH_cc_ (J/g)	X_c_ (%)
PLA	60.1	127.5	4.7	151.1	5.1	0.4
H_90_10	59.9	119.3	20.0	149.3	21.3	1.6
TWG_90_10	60.5	118.3	20.9	149.4	21.9	1.2
H_70_30	59.8	121.8	15.7	149.9	15.8	0.2
TWG_70_30	59.6	121.7	14.9	149.9	15.5	0.9
H_50_50	59.0	126.0	10.4	150.3	10.8	0.9
TWG_50_50	58.8	128.5	7.0	151.3	7.5	1.1

**Table 5:** DMA research results.

Sample	E’_30_ (MPa)	E’_50_ (MPa)	E’_70_ (MPa)	E’_90_ (MPa)	E’_130_ (MPa)	T_g_ (°C)
PLA	2625	2515	28	7	73	69.1
H_90_10	3149	3005	138	17	166	71.2
TWG_90_10	3047	2906	127	21	161	71.2
H_70_30	4243	4111	358	94	508	71.0
TWG_70_30	3984	3837	398	78	470	71.9
H_50_50	5427	5267	1384	309	1088	72.6
TWG_50_50	5716	5520	888	253	733	71.0

**Table 6:** ANOVA test on the effects of the kind of natural filler content on WPC’s mechanical properties (*p*-values).

Property	Filler Content (%)		
	10	30	50
Tensile modulus	0.026138 *	0.042118 *	0.004789 **
Tensile strength	0.000033 **	0.071074 ^ns^	0.007733 **
Impact strength	0.690906 ^ns^	0.743999 ^ns^	0.016076 *

* Significant at 0.01; ** significant at 0.05; ns, nonsignificant at 0.05.
